# Multi-omics changes in quality, metabolites, and microbial communities of De'ang pickled tea across production years

**DOI:** 10.1016/j.fochx.2026.104685

**Published:** 2026-10-25

**Authors:** Lihong Chen, Yunfang Li, Xueqing Dang, Fengmei Lu, Weixing Yang, Zixian Lv, Yumeng Qin, Yi Zhang, Yan Hou

**Affiliations:** a College of Tea Science, Yunnan Agriculture University, Kunming 650201, Yunnan, China; b Key Laboratory of Tropical Plant Resources and Sustainable Use, Xishuangbanna Tropical Botanical Garden, Chinese Academy of Sciences, Menglun, Mengla 666303, Yunnan, China; c Yunnan Defeng Tea Industry Co., Ltd, Mangshi 678400, Yunnan, China; d College of Biological and Food Engineering, Qujing Normal University, Qujing 655011, Yunnan, China; e The Key Laboratory of Agricultural Microbiome of Yunnan Province, Kunming 650201, Yunnan, China

**Keywords:** De'ang pickled tea, Production year, Untargeted metabolomics, Volatile aroma compounds, Bacterial community, Multi-omics correlation

## Abstract

De'ang pickled tea is a traditional fermented tea undergoing complex chemical and microbial transformations during long-term production and storage. This study investigated production-year-related variations (2013–2024) in sensory quality, volatile compounds, metabolites, and bacterial communities using a multi-omics approach. Sensory evaluation revealed differences in aroma, taste, and liquor color among samples. GC–MS identified 1351 volatile compounds, and 23 key aroma-active compounds (rOAV ≥1) were determined as major contributors to characteristic aroma attributes. Untargeted metabolomics identified 2945 metabolites and revealed significant differences in flavonoid-, lipid-, and nitrogen-related metabolic pathways across production years. Major chemical constituents and antioxidant activities varied among samples without consistent temporal trends. 16S rRNA sequencing demonstrated shifts in bacterial diversity and community composition, with Lactiplantibacillus and Klebsiella as dominant genera. Multi-omics correlation analysis suggested associations between microbial taxa and chemical constituents, highlighting coordinated variation in microbial and metabolic profiles across production years.

## 1 Introduction

De'ang pickled tea is a traditional ethnic fermented tea originating from southwestern Yunnan Province, China. It is produced by the De'ang people using fresh leaves of large-leaf tea cultivars, which are tightly packed, sealed, and subjected to spontaneous anaerobic fermentation, followed by long-term storage. De'ang pickled tea is characterized by a pronounced pickled aroma, a refreshing and cooling mouthfeel, and a lingering sweet aftertaste with moderate acidity. Previous studies have suggested that De'ang pickled tea possesses traditional health-promoting properties, including anti-obesity effects, prevention of non-alcoholic fatty liver disease, and a reduced risk of hepatic lipid accumulation. At present, research on De'ang pickled tea has mainly focused on its cultural history, processing technology (Haiyan et al., 2023; Zhou et al., 2025), chemical composition (Hou et al., 2023), microbial characteristics (Mao et al., 2024; Zhang, Liu, et al., 2020), and health-related functions. However, systematic studies addressing quality differences among De'ang pickled teas with different storage years have not yet been reported.

In recent years, increasing attention has been paid to the influence of storage duration on tea quality formation. Existing studies have demonstrated that different storage years can significantly affect the chemical composition, aroma characteristics, and microbial community structure of tea leaves (Yin et al., 2022). Across various tea types, tea polyphenols, catechins, caffeine, amino acids, and flavonoids exhibit pronounced dynamic changes during storage, while microbial succession is considered a key driving force for flavor evolution (Liu et al., 2023; Tian et al., 2013; Wang et al., 2024). Similar production-year-related changes have been reported in Pu-erh tea, a traditionally post-fermented tea produced from sun-dried large-leaf tea. In raw Pu-erh tea, storage time has been shown to influence catechins, gallic acid, and flavonoids, with significant changes in compounds such as EGCG, ECG, GCG, kaempferol, and quercetin (Zhou et al., 2020). Although both Pu-erh tea and De'ang pickled tea undergo fermentation-related transformations, their processing and fermentation characteristics differ, and therefore their production-year-related compositional changes may not follow the same patterns. In addition, multi-omics studies have suggested potential associations between microbial communities and variations in polyphenols, amino acids, and volatile aroma compounds across different production years (Ma et al., 2025; Zhang et al., 2024; Zhang et al., 2025).

Despite these advances, comprehensive studies on De'ang pickled tea produced in different years remain scarce. In particular, integrated analyses combining sensory evaluation, non-volatile metabolites, volatile flavor compounds, and microbial community profiling across production years have not been systematically conducted. Moreover, the relationships between microbial community shifts and metabolite variation across production years remain insufficiently understood.

Therefore, De'ang pickled tea samples produced from 2013 to 2024 under the same processing and storage conditions were collected from a single manufacturer. Sensory evaluation, chemical composition analysis, non-targeted metabolomics, volatile profiling, and 16S rRNA gene sequencing were performed, combined with multivariate statistical and correlation analyses. This study aimed to systematically characterize production-year-associated differences in the sensory quality, chemical composition, volatile profiles, and microbial communities of De'ang pickled tea, and to explore potential associations between microbial composition and metabolite profiles across different production years. The results provide a comprehensive multi-omics framework for understanding quality-related variations in traditional fermented teas across production years.

## 2 Materials and methods

### 2.1 Tea materials and sample preparation

In this study, twelve batches of De'ang pickled tea produced from Yunnan large-leaf tea (*Camellia sinensis*) were collected from Dehong Defeng Tea Co., Ltd., Yunnan Province. The raw materials for each production year were harvested annually between April 20 and May 20. All samples were analyzed in 2025. For each production year, three independent replicate samples were obtained and used for subsequent analyses, including sensory evaluation, electronic nose analysis, determination of major chemical components, in vitro antioxidant activity, volatile flavor analysis, non-targeted metabolomics, and 16S rRNA gene sequencing.

All tea samples were manufactured under standardized industrial conditions according to the same enterprise production protocol. The processing followed the Technical Regulations for De'ang Pickled Tea Processing (DB5331/T35–2021), including withering, fixation, rolling, de-blocking, anaerobic fermentation, and drying. Fixation was performed using a 6CCT-110 frying machine for 20 min, and rolling was conducted using a type-50 tea rolling machine for 25 min. After de-blocking, tea leaves were layered and compacted into ceramic fermentation vessels, sealed, and subjected to anaerobic fermentation for approximately six months under controlled production conditions before drying and final processing. Although all samples were produced following the same enterprise protocol, year-to-year variations in production conditions could not be completely excluded.

After production, all tea samples were stored under standardized and identical conditions provided by the manufacturer to minimize environmental variability among batches. Specifically, the samples were sealed in moisture-proof and food-grade packaging materials and stored in a dedicated warehouse under dark conditions, without exposure to direct light or odor contamination. The storage environment was maintained at ambient room temperature (approximately 20–25 °C) with relatively stable low humidity (approximately 50% relative humidity). All samples were stored in the same facility under identical ventilation and spatial conditions, and no refrigeration or heating was applied during the entire storage period.

### 2.2 Sensory evaluation

Sensory evaluation was conducted in accordance with the Chinese national standard GB/T 23776-2018, Methodology of Sensory Evaluation of Tea, and the local standard DB5331/T 39-2023, Sensory Evaluation Method for De'ang Pickled Tea (Dry Tea). Five professional tea assessors with relevant experience in tea quality evaluation participated in the sensory assessment. The assessors had received relevant training in sensory evaluation and undergone calibration before the formal assessment to ensure consistent application of the sensory descriptors and scoring criteria specified in the standards.

For each sample, 5.0 g of representative tea was weighed and placed in a cylindrical sensory evaluation cup. Boiling water was added at a tea-to-water ratio of 1:50 (*w*/*v*), and the cup was covered and left to infuse for 5 min. The infusion was then decanted into tasting bowls at a consistent rate and in the order of brewing. The liquor color was assessed first, followed by the aroma of the infused leaves and the taste of the tea liquor. A second infusion was subsequently prepared using the same procedure and infusion time of 5 min, after which the liquor color, aroma, taste, and infused leaves were evaluated. The liquor color was primarily assessed from the first infusion, whereas aroma and taste were primarily assessed from the second infusion. Zhenming bottled mineral water (Kunming, China), with a measured pH of 8.8, was used for sensory evaluation. Five sensory attributes were evaluated: dry tea appearance, liquor color, aroma, taste, and infused leaves. The corresponding weighting coefficients for compressed steamed tea were 20%, 10%, 30%, 35%, and 5%, respectively. The total sensory score was calculated by multiplying the score for each attribute by its corresponding weighting coefficient and summing the weighted scores, according to the formula:

Y=A×0.20+B×0.10+C×0.30+D×0.35+E×0.05

where Y represents the total sensory score, and A, B, C, D, and E represent the scores for dry tea appearance, liquor color, aroma, taste, and infused leaves, respectively. The corresponding weighting coefficients were 0.20, 0.10, 0.30, 0.35, and 0.05, respectively. The individual scores assigned by the five assessors were recorded for each sample. The mean and standard deviation (SD) were calculated to summarize the sensory scores, and the results are presented as mean ± SD. The coefficient of variation (CV) was calculated as the SD divided by the mean and expressed as a percentage to characterize the variability in scores among assessors.

Sensory evaluation involved only the assessment of tea products and did not involve clinical intervention or the collection of personal or identifiable information from the assessors. Before the sensory evaluation, all five assessors were informed of the purpose and procedures of the study and provided consent to participate in the sensory evaluation and for the use of their evaluation data for research purposes. The rights, privacy, and confidentiality of all assessors were respected throughout the study, and no personal information, such as names, signatures, or other identifying information, was collected or disclosed. As the study involved only sensory assessment of tea products and did not involve human subjects research requiring institutional review, ethical approval was not required according to the applicable institutional guidelines.

### 2.3 Determination of chemical components

Seventeen target compounds, including gallic acid (GA), gallocatechin (GC), theobromine, epigallocatechin (EGC), catechin (C), caffeine, caffeic acid (CA), epigallocatechin gallate (EGCG), epicatechin (EC), gallocatechin gallate (GCG), epicatechin gallate (ECG), taxifolin, catechin gallate (CG), rutin, myricetin, quercetin, and kaempferol, were quantified by HPLC.

Tea samples were extracted according to the method of Mao et al. (2024) with minor modifications. Briefly, 3.0 g of tea sample was mixed with 70% (*v*/v) methanol-water solution and extracted at 4 °C for 24 h. After centrifugation at 13,800*g* for 10 min at 4 °C, the supernatant was collected and filtered through a 0.45 μm nylon membrane prior to HPLC analysis.

Chromatographic analysis was performed using an Agilent 1200 series HPLC system (Agilent Technologies, Santa Clara, CA, USA) equipped with a diode-array detector (DAD). Separation was achieved using a Poroshell 120 EC-C18 column (100 mm × 4.6 mm, 2.7 μm; Agilent Technologies, Santa Clara, CA, USA) coupled with a Phenomenex C18 guard column (10 mm × 4.6 mm, 5 μm; Phenomenex, Torrance, CA, USA). The mobile phase consisted of solvent A (50 mL/L acetonitrile containing 2.61 mL/L orthophosphoric acid) and solvent B (800 mL/L methanol). Gradient elution was performed as follows: 0–16.0 min, 10–45% B; 16.0–22.0 min, 45–65% B; 22.0–25.0 min, 65% B; 25.0–25.9 min, 65–100% B; 25.9–29.0 min, 100% B; and 29.0–30.0 min, 100–10% B. The flow rate was maintained at 0.8 mL/min, the column temperature was set at 35 °C, and the injection volume was 2 μL. Detection wavelengths were set at 280 nm during 0–16 min and 360 nm during 16–30 min. Compound identification was achieved by comparing retention times and UV spectra with those of authentic standards. Quantification was conducted by using analytical calibration curves (R^2^ > 0.990). Each sample was analyzed in triplicate, and the mean values were used for subsequent statistical analyses.

### 2.4 Colorimeter measurement

Color parameters of both De'ang pickled tea leaves and infusions were measured with a CM-3600d colorimeter (Konica Minolta, Shanghai, China) using the Lab color system. The appearance color of tea samples was measured directly. The tea infusions were prepared according to the national standard (GB/T 23776-2018). Approximately 3.0 g of de'ang pickled tea was sampled, 150 mL of boiling distilled water was added, and after 5 min of steeping, the tea infusion was filtered. The color parameters (L*, a*, b*) were then measured after the filtrate cooled to room temperature. L* indicates brightness, with values ranging from 0 to 100, where higher values represent higher brightness. The a* value represents red-green color, with “+” indicating red and “-” indicating green. The b* value represents yellow-blue color, with “+” indicating yellow and “−” indicating blue. Measurement of each sample was repeated 9 times.

### 2.5 Determination of in vitro antioxidant activities

The in vitro antioxidant activities of De'ang pickled tea extracts were evaluated using DPPH radical scavenging, ABTS radical scavenging, and ferric reducing antioxidant power (FRAP) assays. Tea extracts were prepared as described above for chemical component analysis.

DPPH radical scavenging activity was determined using a DPPH Radical Scavenging Activity Assay Kit (ADS-W-DP001-196, ADS Biotechnology Co., Ltd., China) according to the manufacturer's instructions. Briefly, 150 μL of tea extract was mixed with 150 μL of DPPH working solution and incubated in the dark at room temperature for 30 min. The absorbance was measured at 517 nm using a microplate reader. The DPPH radical scavenging activity was calculated as:

$$\text{DPPH scavenging activity}\;\left(\%\right)=\left[1-\left(\text{Asample}-\text{Acontrol}\right)/\text{Ablank}\right]\times 100$$

where Asample, Acontrol, and Ablank represent the absorbance values of the sample, control, and blank, respectively.

ABTS radical scavenging activity was measured using an ABTS Radical Scavenging Activity Assay Kit (ADS-W-AB001–196, ADS Biotechnology Co., Ltd., China). Briefly, 10 μL of tea extract was mixed with 190 μL of freshly prepared ABTS working solution in a 96-well microplate and incubated in the dark at room temperature for 6 min. Absorbance was recorded at 734 nm, and the scavenging activity was calculated using the same equation as described for the DPPH assay.

The ferric reducing antioxidant power (FRAP) was determined using a Total Antioxidant Capacity (FRAP) Assay Kit (ADS-W-KY005–196, ADS Biotechnology Co., Ltd., China). Briefly, 5 μL of tea extract, 25 μL of distilled water, and 170 μL of freshly prepared FRAP working reagent were mixed in a 96-well microplate and incubated at room temperature for 10 min. Absorbance was measured at 590 nm. Trolox was used as the standard antioxidant, and the FRAP values were expressed as μmol Trolox equivalents per gram dry weight (μmol TE g^−1^ DW).

All measurements were performed in triplicate, and the mean values were used for statistical analysis.

### 2.6 Electronic nose analysis

The volatile profiles of De'ang pickled tea samples were analyzed using a PEN3 electronic nose system (Airsense Analytics GmbH, Schwerin, Germany) equipped with an array of 10 metal oxide semiconductor (MOS) sensors. The sensor array included W1C (aromatic compounds), W5S (nitrogen oxides), W3C (ammonia and aromatic compounds), W6S (hydrides), W5C (short-chain alkanes and aromatic compounds), W1S (methyl compounds), W1W (sulfides), W2S (alcohols, aldehydes, and ketones), W2W (aromatic compounds and organic sulfides), and W3S (long-chain alkanes).

For analysis, 3.0 g of tea sample was placed in a 20 mL sealed headspace vial and equilibrated at 60 °C for 30 min. The headspace gas was then introduced automatically into the sensor chamber for analysis. The sampling flow rate was maintained at 300 mL/min. The measurement time was 60 s, and sensor responses were recorded at 1 s intervals. After each measurement, the system was purged with clean air to restore the sensor baseline.

The stable response values of the sensors were extracted and used for subsequent statistical analyses. Each sample was analyzed in triplicate.

### 2.7 HS-SPME-GC–MS analysis of volatile compounds

Volatile compounds in De'ang pickled tea samples were analyzed using headspace solid-phase microextraction coupled with gas chromatography–mass spectrometry (HS-SPME-GC–MS). Approximately 1.0 g (±5 mg) of tea sample was placed into a 20 mL headspace vial, and 10 μL of 2-octanol (10 mg/L in distilled water) was added as an internal standard.

Volatile compounds were extracted using an automated solid-phase microextraction system (PAL rail system, Agilent Technologies, Santa Clara, CA, USA). The extraction conditions were as follows: incubation temperature, 60 °C; equilibration time, 15 min; extraction time, 30 min; and desorption time, 4 min. GC–MS analysis was performed using an Agilent 7890B gas chromatograph coupled with an Agilent 5977B mass spectrometer (Agilent Technologies, Santa Clara, CA, USA). Separation was carried out on a DB-Wax capillary column (30 m × 0.25 mm i.d., 0.25 μm film thickness). Helium (≥99.999%) was used as the carrier gas at a constant flow rate of 1.0 mL/min in splitless mode, with a purge flow of 3 mL/min. The oven temperature program was set as follows: initial temperature 40 °C (held for 4 min), increased at 5 °C/min to 245 °C, and held for 5 min. The injector, transfer line, ion source, and quadrupole temperatures were 250 °C, 250 °C, 230 °C, and 150 °C, respectively. Electron impact ionization was performed at 70 eV. Mass spectra were acquired in full scan mode over the *m*/*z* range of 20–400 with a solvent delay of 0 min.

Data processing was performed using ChromaTOF 4.3× software (LECO Corporation, St. Joseph, MI, USA), including peak extraction, baseline correction, alignment, deconvolution, peak identification, and peak area integration. Volatile compounds were tentatively identified by comparison of mass spectra with the NIST library.

### 2.8 Relative odor activity value (ROAV) analysis

The relative odor activity value (ROAV) was used to evaluate the contribution of individual volatile compounds to the overall aroma profile and to identify key odor-active compounds. For each compound, the relative percentage of peak area (C%) was calculated based on the mean peak area within each sample group. Only compounds with reported odor threshold values from the literature were included in the analysis. The compound with the highest contribution to the overall aroma was defined as the reference compound, and its ROAV was set to 100 (ROAV_ref). The ROAV of each target compound (ROAV_i) was calculated using the following equation:

$$\text{ROAV}\_i=\left(C\_i/T\_i\right)÷\left(C\_\mathrm{ref}/T\_\mathrm{ref}\right)\times 100$$

where C_i and C_ref represent the relative peak area percentages of the target compound and reference compound, respectively, and T_i and T_ref represent their corresponding odor threshold values.

Compounds with ROAV ≥1 were defined as key aroma-active compounds, while those with 0.1 ≤ ROAV <1 were considered to contribute to aroma modification.

### 2.9 Non-targetedmetabolomic detection of non-volatile compounds in pickled tea samples

Freeze-dried tea samples were ground using a mixer mill. Approximately 50 mg (±5 mg) of each sample was accurately weighed into a centrifuge tube, and 500 μL of 80% ice-cold methanol was added. A small amount of steel balls was added, and the samples were homogenized using a grinder. The samples were then incubated at −20 °C for 30 min to precipitate proteins, followed by centrifugation at 20,000 ×*g* for 10 min at 4 °C. The supernatant was centrifuged again for 5 min, and the resulting supernatant was transferred to a fresh vial for UPLC-HRMS analysis. Quality control (QC) samples were prepared by mixing equal aliquots of the supernatants from all samples. No internal standards were added for absolute quantification.

Chromatographic separation was performed using an ACQUITY UPLC HSS T3 column (100 mm × 2.1 mm, 1.8 μm; Waters). The mobile phase consisted of phase A (5 mmol/L ammonium acetate and 5 mmol/L acetic acid in water) and phase B (acetonitrile). The flow rate was 0.35 mL/min, the injection volume was 4 μL, and the column temperature was maintained at 40 °C.

Metabolites were detected by high-resolution mass spectrometry in both positive and negative electrospray ionization modes. The mass spectrometric data were acquired using full-scan and data-dependent acquisition modes. No external calibration curves or standard addition methods were used for metabolite quantification in the non-targeted metabolomics analysis.

The acquired LC–MS data were preprocessed using XCMS software, including peak picking, peak grouping, retention time correction, second peak grouping, and annotation of isotopes and adducts. The raw data files were converted into mzXML format and subsequently processed using XCMS, CAMERA, and the metaX toolbox implemented in R. Metabolites were annotated using the KEGG and HMDB databases by matching the exact molecular mass (*m*/*z*) of detected ions with database entries. A mass difference of less than 10 ppm between the observed and database values was used as the criterion for metabolite annotation. An in-house fragment spectrum library was also used to further validate metabolite identification.

Statistical analyses were primarily performed using R (v4.2.2). Data filtering, K-nearest neighbor (KNN) imputation, and probabilistic quotient normalization (PQN) were performed prior to statistical analysis. Principal component analysis (PCA) and partial least squares discriminant analysis (PLS-DA) were conducted using the ropls packages. KEGG pathway enrichment analysis was performed using the hypergeometric test, with *P*-value <0.05 considered statistically significant.

### 2.10 DNA extraction and 16S rRNA gene sequencing analysis

Genomic DNA was extracted from pickled tea samples using the CTAB method (Nobleryder, China) according to the manufacturer's instructions. DNA quality was evaluated by 1% agarose gel electrophoresis in 1× TAE buffer. The bacterial 16S rRNA gene V3–V4 regions were amplified using the primer pair 341F (5′-CCTAYGGGRBGCASCAG-3′) and 806R (5′-GGACTACNNGGGTATCTAAT-3′). PCR amplification was performed in a 25 μL reaction system containing 15 μL Phusion High-Fidelity PCR Master Mix, 0.2 μM of each primer, and approximately 10 ng of template DNA. Thermal cycling conditions were as follows: initial denaturation at 98 °C for 1 min; 30 cycles of denaturation at 98 °C for 10 s, annealing at 50 °C for 30 s, and extension at 72 °C for 30 s; followed by a final extension at 72 °C for 5 min. PCR products were purified using magnetic bead-based purification methods and verified by 1% agarose gel electrophoresis. Amplicons were quantified, and equimolar amounts were pooled for library construction with index adapters.

Sequencing libraries were assessed for quality using Qubit fluorometric quantification, qPCR, and fragment size distribution analysis using a Bioanalyzer system. Qualified libraries were pooled in equimolar amounts and sequenced on an Illumina paired-end sequencing platform. Raw sequencing data were subjected to quality filtering, chimera removal, and sequence denoising prior to downstream microbial community analysis.

### 2.11 Bioinformatics and statistical analysis

Principal component analysis (PCA) were performed using SIMCA-P software (version 18.0.1). Heatmaps of metabolite profiles were generated using the “pheatmap” package (version 1.0.12) in RStudio (version 4.4.2). Venn diagrams were constructed using the “VennDiagram” package, and UpSet plots were generated using the “UpSetR” package. Radar charts were produced using the “fmsb” package (version 0.7.1). K-means clustering analysis and visualization were performed using the “TCseq” package (version 1.30.0). Bar plots and KEGG enrichment bubble plots were generated using the “ggplot2” package (version 3.5.1).

The correlations among metabolites, microbial taxa, and sensory scores were calculated using the “psych” package (version 2.4.6.26) in R. Statistical significance of correlations was evaluated based on *p*-values, with *p* < 0.05 considered statistically significant. Correlation coefficients (r) were used to describe the strength and direction of associations, where |r| > 0.8 indicated strong positive or negative correlations. For network visualization, only statistically significant correlations (*p* < 0.05) with |r| > 0.8 were used to construct correlation networks in Cytoscape (version 3.9.1).

Significant differences in metabolites and sensory scores among groups were evaluated using one-way analysis of variance (ANOVA) followed by Tukey's post hoc test or two-sample *t*-test, with *p* < 0.05 considered statistically significant.

## 3 Results and discussion

### 3.1 Sensory evaluation of De'ang pickled tea of different production years

To investigate the flavor differences of De'ang pickled tea from different production years, a trained sensory panel conducted traditional sensory evaluations on samples collected from 2013 to 2024, focusing on appearance, liquor color, aroma, taste, and infused leaf quality. According to the evaluations provided by professional tea assessors (Fig. 1A, Table S1), the appearance and infused leaves of all samples showed no obvious differences across production years, consistently presenting regular strip shapes with dark green to brownish coloration. The infused leaves were generally soft and relatively uniform, with slight variations in leaf integrity and color intensity among samples. In terms of aroma, the 2016 sample exhibited relatively weak acidity and a mixed scent profile, whereas the other samples showed more pronounced acidic aroma characteristics, with the 2024 sample presenting a relatively persistent acidic note. Regarding taste, samples from 2021 to 2023 exhibited slight astringency; in contrast, samples from other production years showed comparatively lower astringency and a more balanced taste profile with improved harmony between acidity and sweetness. Comprehensive sensory scores ranged from 80 to 96 among samples from different production years (Table S1), further indicating sensory variation among the samples.

**Fig. 1:**
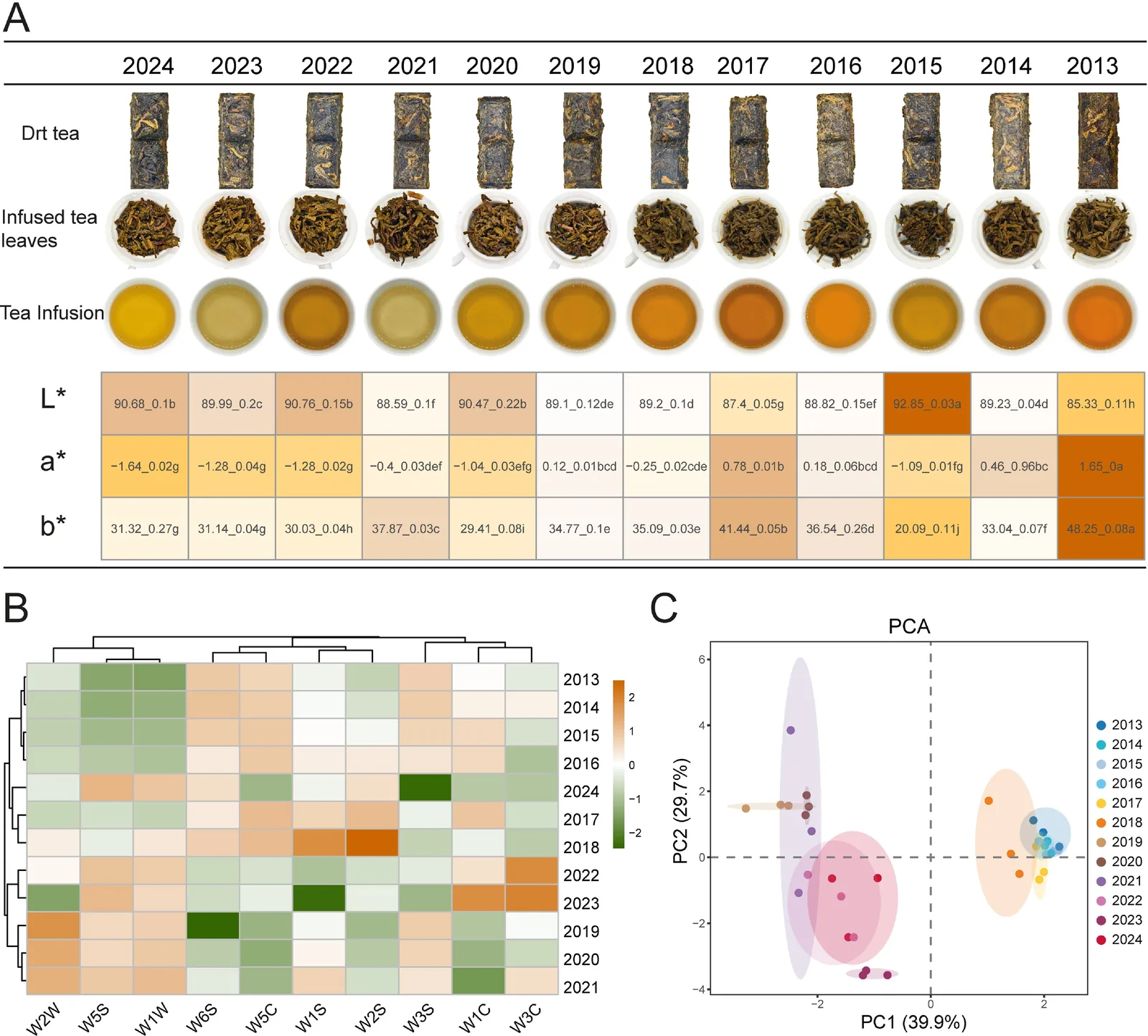
Sensory evaluation and electronic nose analysis of De'ang pickled tea across different production years. (A) Tea appearance, infusion, and infused leaves of De'ang pickled tea from 2013 to 2024, along with tea liquor color parameters (L* for lightness, a* for red–green, and b* for yellow–blue). (B) Heatmap of electronic nose sensor responses across samples from different production years (2013–2024). (C) Principal component analysis (PCA) score plot based on electronic nose response data. (For interpretation of the references to color in this figure legend, the reader is referred to the web version of this article.)

Liquor color is one of the key indicators of tea quality. Analysis of tea liquor color parameters across different production years revealed significant differences in brightness (L), red–green difference (a), and yellow–blue difference (b) (Fig. 1A). The 2015 tea liquor exhibited the highest brightness, while 2013 had the lowest. For red–green difference (a), the 2013 liquor showed the strongest red tone, 2016 appeared greener, and 2024 and 2023 also skewed green, with the other years relatively balanced. Regarding yellow–blue difference (b), 2013 liquor displayed the strongest yellow tone, followed by 2017. Overall, tea liquor color varied among production years, ranging from bright yellow to darker reddish-yellow hues.

### 3.2 Electronic nose analysis of De'ang pickled tea from different production years

Principal component analysis (PCA) based on electronic nose data (Fig. 1B) showed that PC1 and PC2 explained 39.9% and 29.7% of the total variance, respectively. Along the PC1 axis, samples from 2013 to 2018 were mainly distributed on the positive side, whereas samples from 2019 to 2024 were located on the negative side, indicating a certain separation trend in volatile response characteristics among samples from different production years.

To further explore the basis of this separation, the responses of individual electronic nose sensors were analyzed (Fig. 1C). The results showed that sensors such as W5S, W1W, W2W, and W3C exhibited relatively higher responses, whereas W6S and W1S showed generally lower and more stable responses across samples. Differences in sensor response patterns were observed among production years, with higher responses of W5S, W1W, and W2W in samples from 2019 to 2024, while relatively higher responses of W6S, W3S, and W5C were observed in samples from 2013 to 2016. These results indicate variations in volatile response characteristics among samples from different production years. To further characterize the volatile composition underlying these differences, GC–MS analysis was subsequently performed.

### 3.3 Changes in major chemical components of De'ang pickled tea during long-term storage

To investigate the chemical basis underlying sensory differences among De'ang pickled tea samples from different production years, 18 major chemical constituents, including catechins, caffeine, theobromine, and flavonoids, were systematically analyzed (Table S1). Catechins, the most important class of tea polyphenols, contribute significantly to bitterness and astringency and are key components influencing tea liquor color, aroma, and taste quality (Ye et al., 2022). Among catechins, epigallocatechin (EGC) was the predominant monomer (0.166–0.748 mg/g), but showed considerable variation across production years without a consistent pattern. EGCG and GCG were present at relatively low levels with limited variation. ECG exhibited pronounced differences among samples, reaching the highest level in 2023 (0.184 ± 0.006 mg/g) and decreasing sharply in 2024 (0.018 ± 0.004 mg/g). EC remained relatively abundant across all samples and reached the highest value in 2024 (0.389 ± 0.019 mg/g). GC also varied among production years, with relatively higher levels in 2014–2016 and 2024, and lower levels in 2013 and 2023.

In the alkaloid fraction, caffeine was the dominant compound (0.853–2.301 mg/g), with the highest level observed in the 2013 sample (2.301 ± 0.612 mg/g), while the remaining years showed relatively comparable levels (approximately 0.85–1.38 mg/g). Similar trends have been reported in white, green, and black teas (Cheng et al., 2021; Dai et al., 2020; Xu et al., 2019). Theobromine was present at trace levels (0–0.009 mg/g) and showed negligible variation among years. Gallic acid (GA) remained at low levels (0.006–0.032 mg/g) with no consistent variation across production years.

In the flavonol group, rutin, myricetin, quercetin, and kaempferol were present at relatively low levels but exhibited noticeable differences among production years. Rutin reached its highest level in 2013 (0.066 ± 0.011 mg/g), was not detected in 2015, and remained low in the other samples (0.018–0.057 mg/g). Myricetin ranged from 0.012 to 0.032 mg/g, with the highest concentration observed in 2013 and lower levels detected in 2019, 2022, and 2024. Quercetin showed relatively minor variation across production years (0.013–0.025 mg/g), whereas kaempferol varied moderately among samples, with higher levels observed in 2015, 2017, and 2024 (0.035–0.038 mg/g) and lower levels in 2013, 2021, and 2023 (0.013–0.023 mg/g).

Overall, the major chemical constituents of De'ang pickled tea remained relatively stable across different production years, whereas some catechins and flavonols showed considerable inter-annual variation, indicating compositional heterogeneity among samples from different years.

### 3.4 In vitro antioxidant activities of De'ang pickled tea samples from different production years

The antioxidant activities of De'ang pickled tea samples from different production years (2013–2024) were evaluated using FRAP, DPPH, and ABTS assays. FRAP values ranged from 2.784 ± 0.217 to 7.265 ± 0.397, with the lowest level observed in 2014 and higher values detected in 2021–2024 (6.788–7.265) (Fig. 2A). ABTS scavenging activity ranged from 36.954% to 60.174%, with the lowest value observed in 2016 and relatively higher values in 2018 and 2024(Fig. 2B). DPPH radical scavenging activity varied from 56.961% to 73.603%, reaching the highest value in 2024 and the lowest in 2015(Fig. 2C). Overall, the three antioxidant assays exhibited variation among production-year samples without a consistent monotonic pattern. The variation in antioxidant activity among De'ang pickled tea samples from different production years may be partly related to differences in their phenolic composition observed among these samples. Tea catechins, including EGC, EGCG, ECG, EC, GC, and GCG, are well recognized for their radical-scavenging and reducing activities and may therefore contribute to the antioxidant capacity observed in the present study (Kanlayavattanakul et al., 2024; Li et al., 2023). Other phenolic compounds detected in the samples, such as rutin, myricetin, quercetin, kaempferol, and gallic acid, may also contribute to this activity through their antioxidant properties (Ma et al., 2022). Thus, the antioxidant capacity of De'ang pickled tea is likely to arise from the combined effects of multiple phenolic constituents rather than from a single compound. Given the complex chemical composition of tea, however, the relative contribution of individual compounds cannot be determined from the present data alone.

**Fig. 2:**
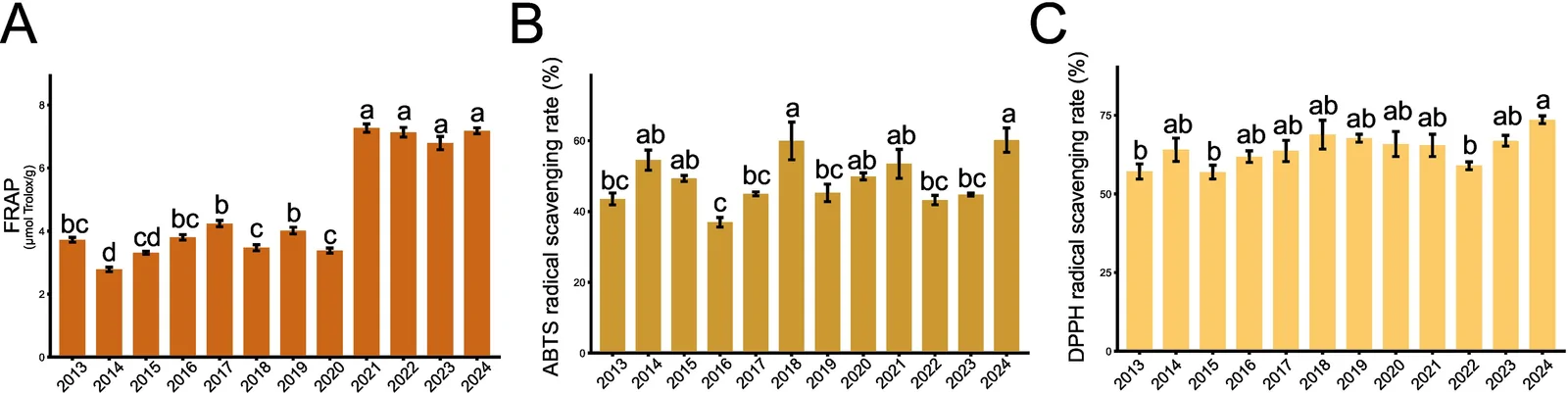
Antioxidant capacity of De'ang pickled tea across different production years (2013–2024) and its correlation with major chemical components. (A) FRAP assay. (B) ABTS assay. (C) DPPH assay.

The fluctuations in antioxidant activity among samples from different production years may be associated with differences in microbial activity during fermentation and variations in storage conditions. Previous studies have shown that microbial fermentation can alter the composition and content of tea phenolic compounds, accompanied by changes in antioxidant activity (Hu et al., 2022; Wang et al., 2021). In addition, storage temperature may affect the stability of catechins and other phenolic compounds, thereby influencing the antioxidant activity of tea (Kim et al., 2020). Differences in fermentation dynamics and storage conditions may therefore lead to varying degrees of chemical transformation among production-year samples, potentially explaining the observed fluctuations rather than a consistent temporal trend. However, the relative contributions of these factors to the observed differences remain to be clarified.

Taken together, the results demonstrate that De'ang pickled tea exhibits in vitro antioxidant activity, although the activity varies among production years. Tea catechins and other polyphenols are widely recognized for their antioxidant properties and other biological activities (Luo et al., 2024; Samynathan et al., 2023). These findings highlight the potential functional value of De'ang pickled tea and provide a basis for further investigation of the factors underlying the variation in antioxidant activity among production-year samples.

### 3.5 Metabolomic profiling of De'ang pickled tea from different

To investigate compositional differences among De'ang pickled tea samples from different production years (2013–2024), a non-targeted metabolomics approach was employed. After data processing and metabolite annotation, a total of 2945 metabolites were identified in both positive and negative ionization modes. These metabolites were mainly classified into lipids and lipid-like molecules (850), organoheterocyclic compounds (642), phenylpropanoids and polyketides (415), benzenoids (387), organic acids and derivatives (285), and organic oxygen compounds (149) (Fig. 3A, Table S3). The relative abundance of metabolites varied across production years, with generally higher signal intensities observed in samples from 2023 to 2024 compared with earlier production years.

**Fig. 3:**
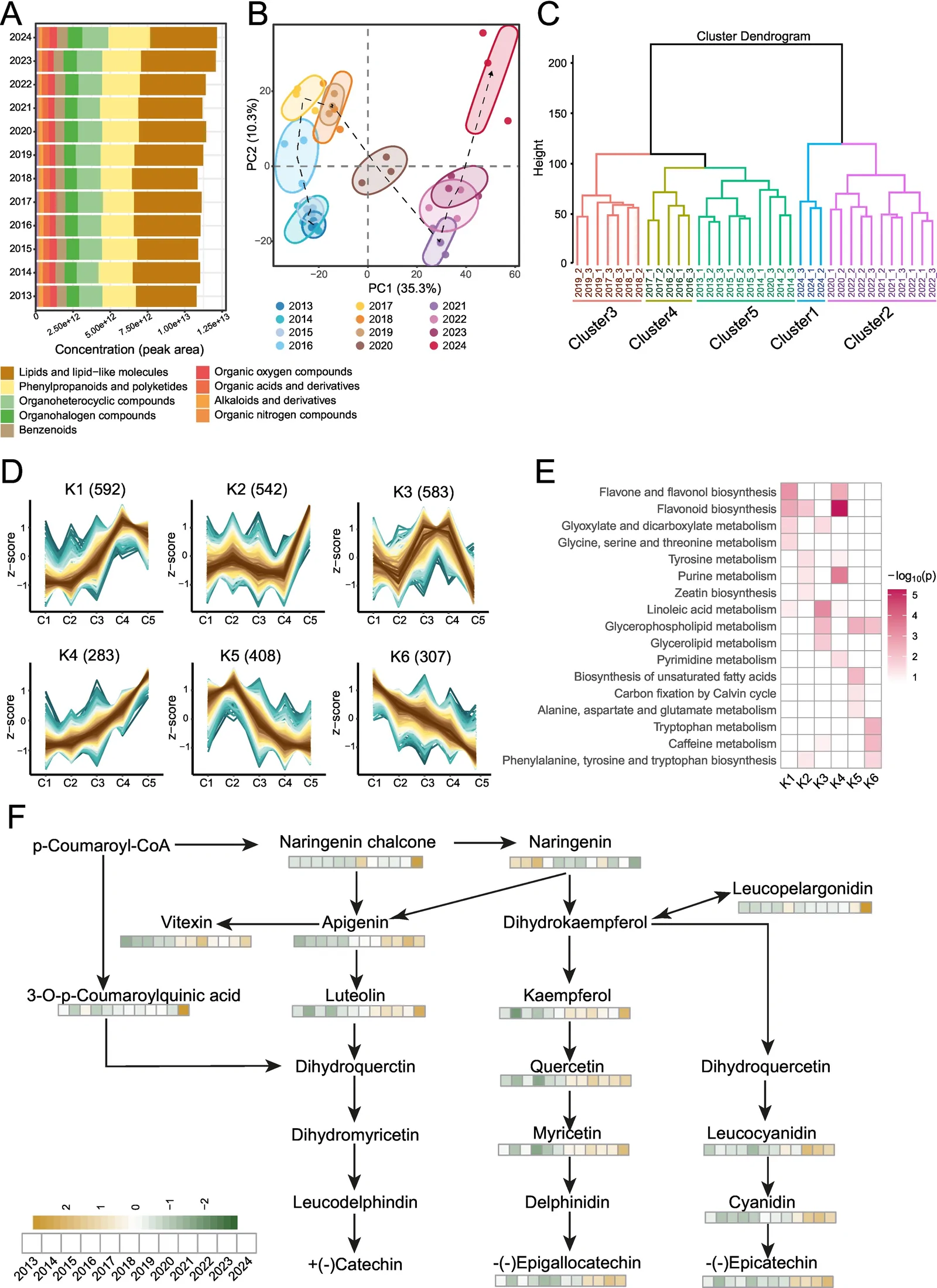
Untargeted metabolomic profiling of De'ang pickled tea from different production years (2013–2024). (A) Classification and relative abundance distribution of metabolites across samples from different production years. (B) Principal component analysis (PCA) of tea samples based on production year. (C) Hierarchical clustering-based sample grouping. (D) k-means clustering-based metabolite trend analysis across production years. (E) KEGG pathway enrichment analysis of metabolites in each k-means cluster. (F) Flavonoid biosynthesis pathway showing the distribution of selected flavonoid-related metabolites across different production years.

PCA revealed a clear separation among samples according to production year, indicating substantial compositional differences across different production years (Fig. 3B). A relatively pronounced shift along PC1 was observed between the 2019 and 2021 samples. Based on hierarchical clustering analysis, samples were classified into five production-year stages: Cluster 1 (2024), Cluster 2 (2020−2023), Cluster 3 (2018–2019), Cluster 4 (2016–2017), and Cluster 5 (2013–2015), showing a clear year-associated grouping pattern (Fig. 3C). PCA exhibited a grouping trend broadly consistent with hierarchical clustering, further supporting the production-year-associated segregation of samples.

K-means clustering further identified six distinct metabolite abundance patterns (K1–K6) across production years (Fig. 3D). K1, K2, and K4 exhibited relatively higher abundance in recent production-year samples (2021–2024), whereas lower levels were observed in earlier production years (2013–2018). K3 showed fluctuating abundance patterns without a clear temporal trend. In contrast, K5 and K6 displayed relatively higher abundance in earlier production-year samples compared with more recent ones. KEGG enrichment analysis indicated that metabolites within different clusters were associated with distinct metabolic pathways (Fig. 3E). K1, K2, and K4 were significantly enriched in flavonoid biosynthesis, suggesting that flavonoid-related metabolites contributed substantially to compositional variation among production years. K3 and K5 were mainly associated with lipid metabolism, including fatty acid and glycerophospholipid pathways, whereas K6 was enriched in nitrogen-containing compound metabolism, such as caffeine-related and aromatic amino acid-associated pathways.

Flavonoid-related metabolites were further examined in the context of the flavonoid biosynthesis pathway to characterize their distribution across De'ang pickled tea samples from different production years (Fig. 3F). Naringenin chalcone showed relatively higher abundance in the 2024 samples, whereas naringenin was more abundant in samples from C5 (2013–2015). Core flavonoid backbone compounds, including apigenin, quercetin, and kaempferol, exhibited relatively higher abundance in mid-production-year samples (C3–C4, 2016–2019). In addition, glycosylated flavonoids, such as quercetin-3-O-rhamnoside and kaempferol derivatives, showed variable abundance across production years without a consistent directional pattern. Catechin-related compounds and their precursors, including (+)-taxifolin, leucocyanidin, leucopelargonidin, epicatechin, and (−)-epigallocatechin, generally exhibited higher relative abundance in samples from C1–C2 (2020–2024) compared with earlier production years.

Overall, these results demonstrate clear differences in the metabolite profiles of De'ang pickled tea across production years, with flavonoid-related compounds representing an important source of compositional differentiation. Changes in flavonoids and catechin-related metabolites may be associated with important taste attributes such as bitterness and astringency, as these compounds are recognized as important contributors to tea taste (Zhang, Cao, et al., 2020). Variations in other metabolite groups, including lipids (Huang et al., 2024) and nitrogen-containing compounds, may also be associated with differences in flavor and other quality-related characteristics. Collectively, the differences in metabolite composition observed across production years may help explain part of the variation in the quality characteristics of De'ang pickled tea. These differences reflect the overall chemical characteristics of individual production years rather than a simple linear change with production year. Untargeted metabolomics revealed changes in flavonoid-related metabolites and the enrichment of associated metabolic pathways among De'ang pickled tea samples from different production years, providing insights into their metabolic compositional differences. However, relative peak intensities may be influenced by ionization efficiency and matrix effects, and pathway enrichment does not directly establish changes in pathway activity or metabolic flux. Targeted quantitative analysis of key metabolites would further validate these findings.

### 3.6 Analysis of volatile flavor compounds in De'ang pickled tea across different production years

A total of 1351 volatile compounds were identified in De'ang pickled tea samples from different production years (2013–2024) by GC–MS, including hydrocarbons (128), alcohols (17), heterocyclic compounds (105), terpenes (99), esters (91), acids (83), phenols (75), and other compounds (62). Fig. 4A shows variations in the contents of different volatile compound classes among samples from different production years. In terms of percentage composition (Fig. 4B), phenols (31–42%) and alcohols (12–23%) were the predominant volatile classes across all samples, followed by esters (8–14%) and terpenes (8–13%), whereas the remaining classes each accounted for less than 10% of the total volatile compounds. The relative abundances of major volatile classes varied among production years (Fig. 4B,Table S4). Although phenols remained the dominant volatile class in all samples, noticeable differences were observed in the proportions of alcohols, esters, terpenes, acids, and heterocyclic compounds, indicating production year-dependent variation in volatile composition.

**Fig. 4:**
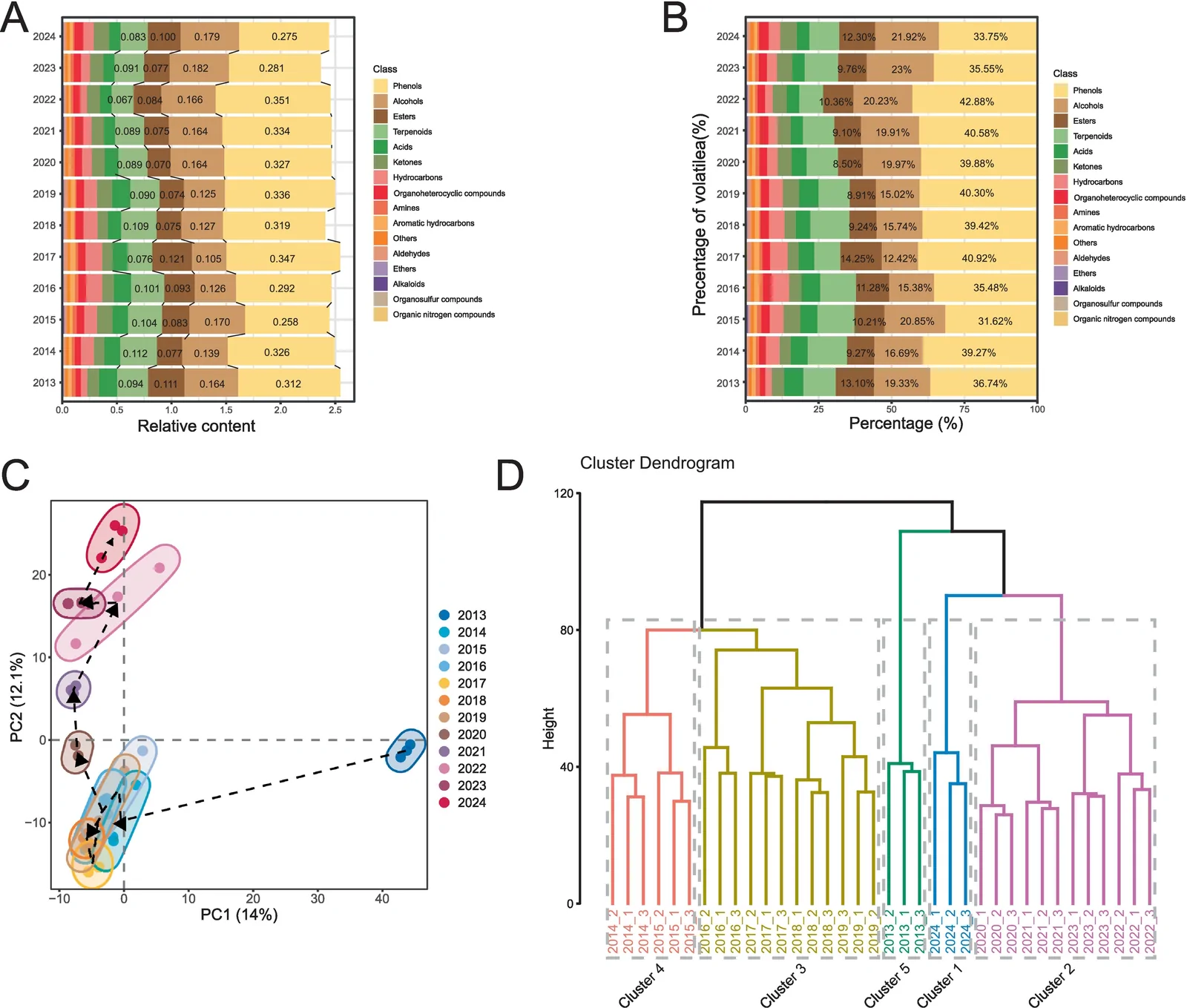
Analysis of volatile flavor compounds in De'ang pickled tea from different production years (2013–2024). (A) Contents of major volatile compound classes among production years. (B) Percentage composition of volatile compound classes across samples from different production years. (C) Principal component analysis (PCA) of volatile profiles based on GC–MS data. (D) Hierarchical clustering analysis.

PCA based on GC–MS data revealed clear separation among samples from different production years (Fig. 4C). PC1 and PC2 explained 14.0% and 12.1% of the total variance, respectively. Samples from 2013 were clearly separated from the other groups, while those from 2014 to 2019 clustered closely. Samples from 2020 exhibited an intermediate distribution, whereas samples from 2021 to 2024 formed a distinct group. Hierarchical clustering analysis further confirmed the sample relationships (Fig. 4D). Replicates clustered tightly within each production year, indicating good analytical reproducibility. Based on the clustering pattern, samples were divided into five clusters: Cluster 1 (2024), Cluster 2 (2020–2023), Cluster 3 (2016–2019), Cluster 4 (2014–2015) and Cluster 5 (2013). The clustering results were consistent with PCA, highlighting that production year is closely associated with variation in volatile profiles. The distinct position of the 2013 samples along PC1 may reflect differences in the initial raw material characteristics, such as those associated with climatic conditions or harvesting time, in addition to possible storage-related changes. However, the available data do not allow these factors to be distinguished independently.

### 3.7 Calculation of relative odor activity value (rOAV) and identification of characteristic flavor compounds

The aroma profile of tea is determined by the combined effects of volatile compound composition and their relative concentrations. The relative odor activity value (rOAV) is commonly used to evaluate the potential contribution of individual volatile compounds to overall aroma perception (Song et al., 2025). Compounds with rOAV ≥1 are generally considered to contribute significantly to aroma characteristics (Jian et al., 2024; Zhai et al., 2022). In this study, 23 key aroma compounds (rOAV ≥1) were identified across samples from different production years, including aldehydes, phenols, alcohols, and ketones (Fig. 5A). Representative compounds such as linalool, benzaldehyde, 2-methylbutanal, methyl salicylate, 1-penten-3-one, (Z)-4-heptenal, and 1-octen-3-ol were consistently detected as important contributors to tea aroma profiles (Liu et al., 2024; Zhai et al., 2022). Based on rOAV values, a flavor wheel was constructed to visualize the aroma characteristics of De'ang pickled tea samples from different production years (Fig. 5B). Variations in rOAV values of key aroma compounds were observed among production years. For example, 1-octen-3-one, associated with mushroom and herbal notes (Feng et al., 2022), showed lower rOAV in 2013 compared with later years. Benzyl alcohol exhibited relatively higher rOAV in several production years including 2013, 2014, 2015, and 2023, indicating variation in floral-related aroma contribution among samples. Some aldehydes such as 4-Heptenal (Hong et al., 2022; Jin et al., 2024; Wang, Li, et al., 2022) and (Z)-2,6-Nonadienal (Lau et al., 2018; Liu et al., 2022) are associated with fresh notes, exhibited higher rOAV values in 2013. Methyl salicylate peaked at 1.75 in 2015, enhancing minty and floral notes (Huang et al., 2023). Linalool also showed higher rOAV in several production years compared with the 2024 sample. Studies show linalool and methyl salicylate increase with Wuyi Rock Tea storage (Cui et al., 2024), but decrease in white tea with prolonged storage (Ma et al., 2023). In the 2016 sample, Styrene, Hexanoic acid, 2-pentyl-Furan, p-Cresol, and Eucalyptol rOAVs peaked. 2-Methylbutanal (Butanal, 2-methyl-) was low in T13 (1.62), gradually increased to T16 (6.32), then slightly decreased but remained above initial levels, indicating storage enhances its aroma contribution.

**Fig. 5:**
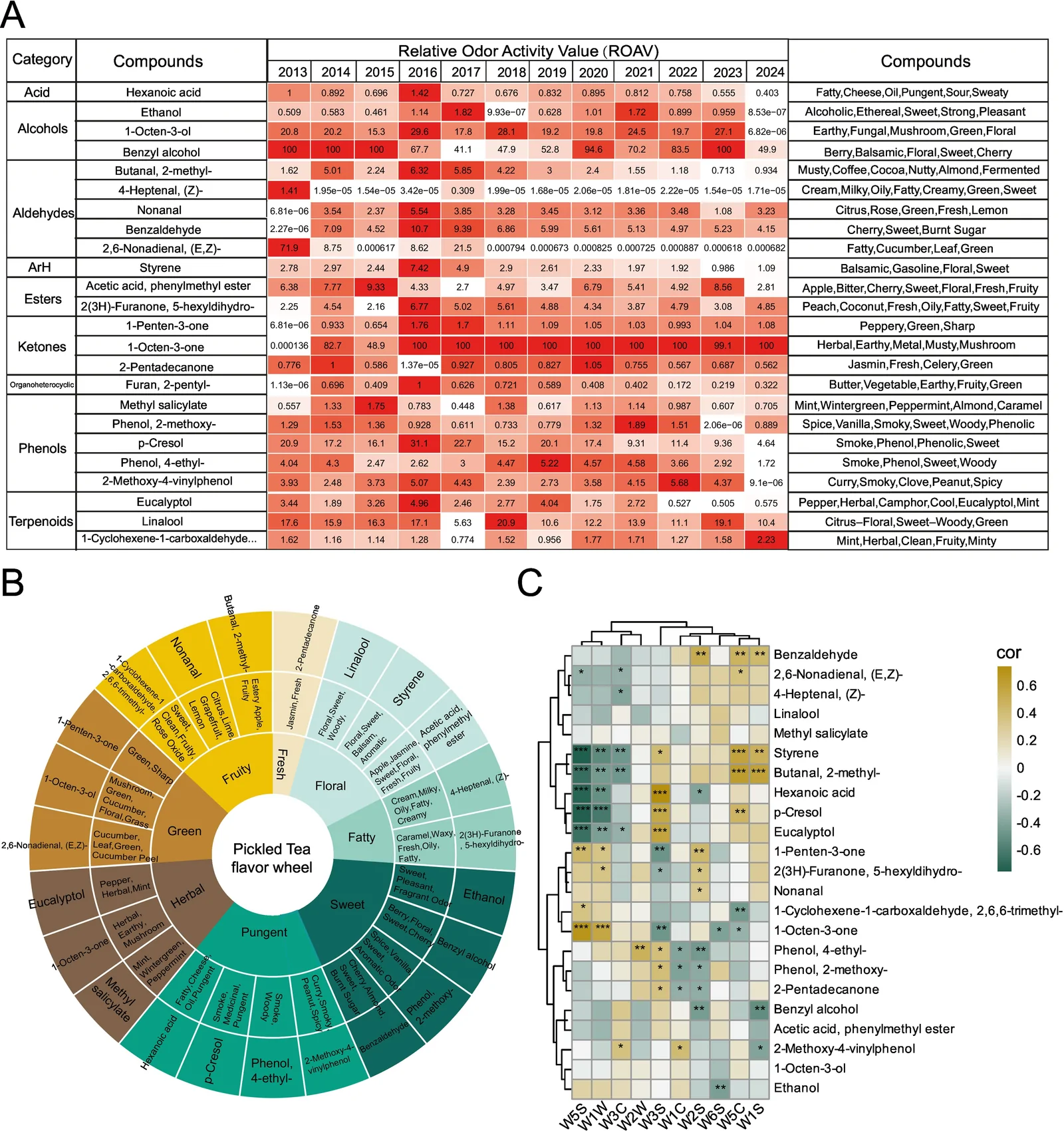
Analysis of characteristic aroma compounds in De'ang pickled tea samples from different production years. (A) Relative odor activity values (rOAVs) and odor descriptions of volatile compounds. (B) Flavor wheel of characteristic volatile compounds. (C) Spearman correlation analysis between electronic nose sensor responses and the relative abundances of 23 key volatile compounds.

To investigate the relationships between electronic nose responses and key aroma compounds, Spearman correlation analysis was performed between sensor responses and the relative abundances of the 23 key volatile compounds (Fig. 5C). Using *P* < 0.05 as the significance threshold, the W3S sensor response was positively correlated with hexanoic acid (*r* = 0.744), p-cresol (*r* = 0.564), and eucalyptol (*r* = 0.595), but negatively correlated with 1-octen-3-one (*r* = −0.468) and 1-penten-3-one (*r* = −0.475). The W5C and W1S sensor responses were positively correlated with 2-methylbutanal (*r* = 0.639 and 0.598, respectively), whereas the W2S sensor response was positively correlated with benzaldehyde (*r* = 0.523).

Overall, the rOAV analysis revealed differences in the potential aroma contributions of key volatile compounds among De'ang pickled tea samples from different production years. Together, the rOAV and electronic nose analyses provided complementary information on the aroma characteristics of samples from different production years.

### 3.8 Bacterial community changes in De'ang pickled tea across different production years

To investigate differences in microbial community composition among De'ang pickled tea samples from different production years (2013–2024), 16S rRNA sequencing was performed to characterize bacterial community structure. The α-diversity indices, including Observed_features, Shannon, Simpson, and Pielou, are shown in Fig. 6A. Overall, bacterial richness (Observed_features) tended to be lower in samples from recent production years (2021–2024), while higher values were observed in earlier production years (2013–2018). Diversity indices (Shannon, Simpson, and Pielou) showed similar variation patterns across production years, suggesting differences in bacterial community diversity among production-year groups. β-diversity analysis using NMDS further revealed clear differences in bacterial community structure among samples from different production years (Fig. 6B). Samples from 2020 to 2024 tended to cluster more closely, whereas samples from 2013 to 2019 showed a more dispersed distribution, indicating a shift in community composition among production-year groups.

**Fig. 6:**
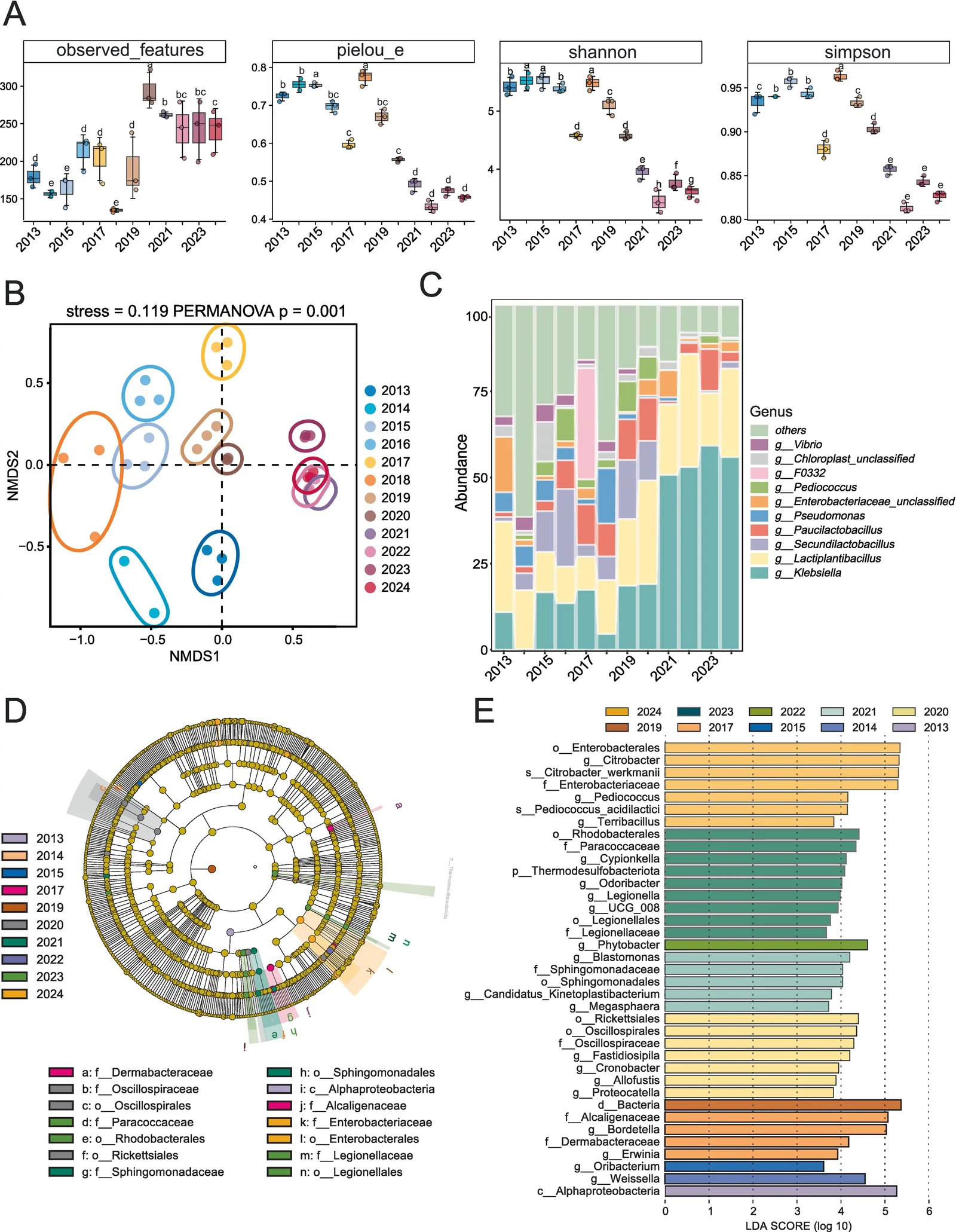
Bacterial community analysis of De'ang pickled tea across different storage years. (A) α-diversity of the microbial community. (B) β-diversity of bacterial communities across different storage years assessed by non-metric multidimensional scaling (NMDS). (C) Relative abundance changes of dominant genera at the genus level across storage years. (D–E) LEfSe analysis.

At the genus level, *Lactiplantibacillus* and *Klebsiella* were identified as dominant taxa across all samples (Fig. 6C). *Klebsiella* showed relatively higher abundance in recent production years (2021–2024), whereas lower levels were observed in earlier production years (2013–2018). The abundance of *Lactiplantibacillus* decreased from 2013 to 2017, partially recovered in 2018–2019, increased markedly in 2020, reached a second peak in 2022, declined in 2023, and rose again in 2024. Despite these fluctuations, *Lactiplantibacillus* has been previously reported as a core and relatively stable genus in De'ang pickled tea fermentation (Cao et al., 2019; Li et al., 2023; Zhang et al., 2023). Importantly, the in vitro antioxidant assays (FRAP, DPPH, and ABTS) showed relatively higher antioxidant capacities in several recent production-year samples (2021–2024). In Miang tea and sour tea fermentations, lactic acid bacteria, particularly *Lactiplantibacillus plantarum*, have been demonstrated to be closely associated with enhanced antioxidant capacity, partly through esterase activities (e.g., TanaA and TanB) that hydrolyze phenolic ester bonds and promote the formation of antioxidant-related compounds such as gallic acid (Curiel et al., 2009; Jiménez et al., 2014; Landete et al., 2021). Overall, these findings suggest that variations in antioxidant activity across production years may be associated, at least in part, with dynamic changes in the abundance of *Lactiplantibacillus*. Although *Klebsiella* was identified as a dominant genus in De'ang pickled tea, some species within this genus are opportunistic pathogens and may pose food safety concerns if pathogenic strains are present in the product. However, 16S rRNA gene sequencing in this study only enabled genus-level identification and could not determine the species identity or pathogenic potential of the detected bacteria. Further species-level identification and safety assessment are therefore needed to evaluate the potential food safety implications.

LEfSe analysis identified taxonomic biomarkers across production-year groups (*p* < 0.05, LDA > 3), indicating clear production-year-dependent shifts in microbial composition (Fig. 6D-E). These taxa may serve as potential indicators for distinguishing different production-year groups.

Overall, bacterial community composition varied among different production-year groups, characterized by shifts in dominant taxa and diversity patterns, while maintaining a relatively stable core microbiota. A compositional transition was observed around the 2020 production-year group based on NMDS and PCoA results. Similar patterns of microbial community variation across production-year or storage-age gradients have been reported in other fermented tea systems, including Bulang pickled tea, Liubao tea, and Tieguanyin tea (Liang et al., 2025; Xu et al., 2025; Yang et al., 2023).

### 3.9 Multi-omics correlation analysis

To explore potential associations between microbial communities and chemical profiles in De'ang pickled tea samples from different production years, correlation networks were constructed based on bacterial genera, volatile compounds, major chemical constituents, and non-volatile metabolites (Fig. 7A–B).

**Fig. 7:**
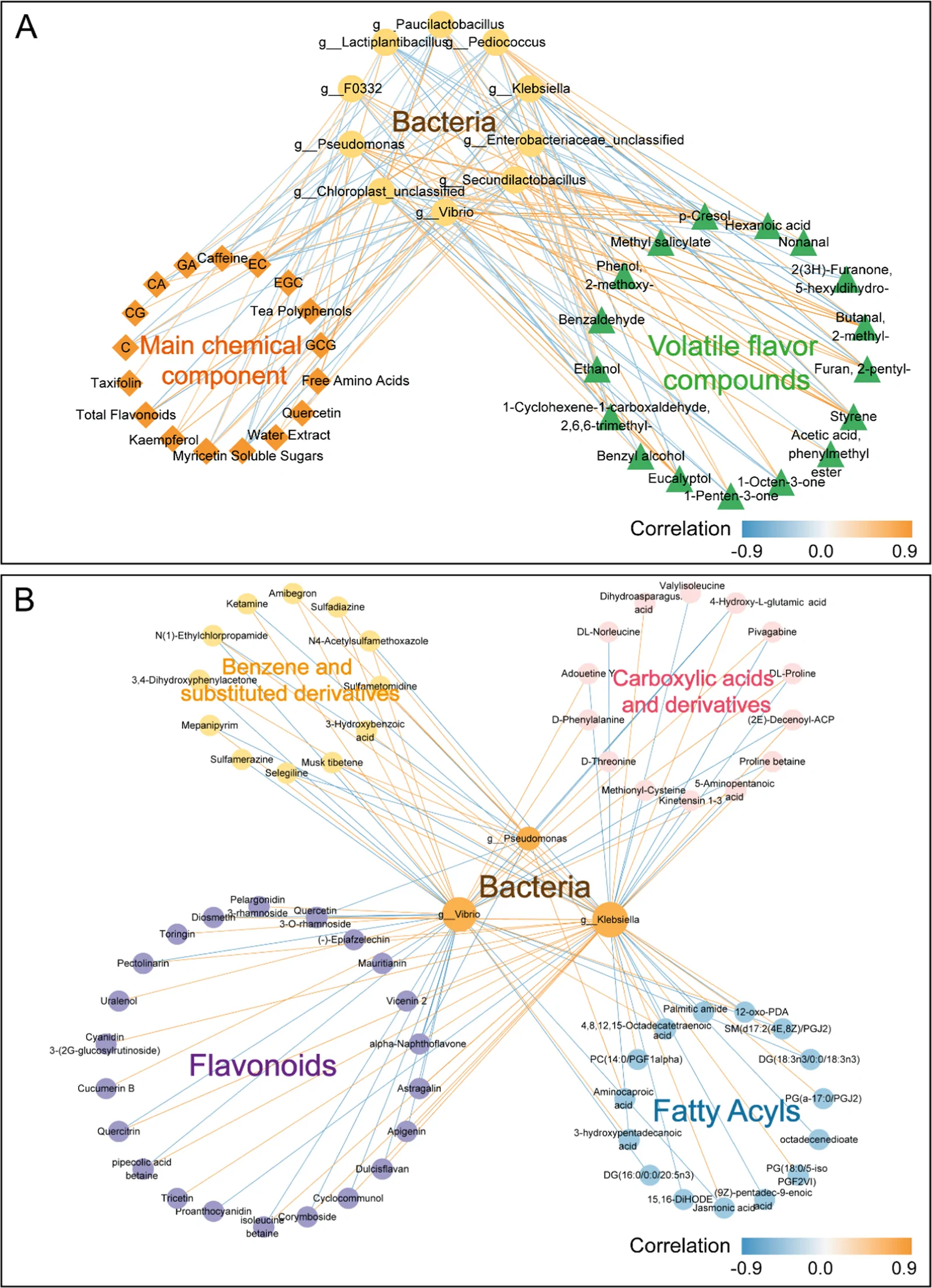
Multi-omics correlation network analysis. (A) Multi-omics correlation network of “volatile flavor compounds / major chemical components – microbes” based on Spearman correlation coefficients (|r| ≥ 0.8, *p* ≤ 0.05). (B) Non-volatile metabolite–microbe correlation network based on Spearman correlation coefficients (|r| ≥ 0.8, *p* ≤ 0.05).

The correlation network between microorganisms and volatile compounds (Fig. 7A) showed that several bacterial genera were significantly associated with characteristic aroma compounds. For example, Vibrio, Pseudomonas, and Secundilactobacillus exhibited positive correlations with Styrene, p-Cresol, Eucalyptol, and 2-Pentylfuran, whereas Klebsiella and Lactiplantibacillus showed correlations with compounds such as 2-Methylbutanal, Benzaldehyde, and Hexanoic acid. In addition, several genera were associated with major chemical constituents, including catechins, flavonoids, caffeine, and antioxidant activity indices. Previous research has shown that fermentation with *Lactiplantibacillus plantarum* can alter the volatile profile of tea-based beverages (Li et al., 2022), suggesting that microbial activity may contribute to aroma variation. However, whether this genus directly produced or transformed the specific volatile compounds identified here remains unclear.

The non-volatile metabolite–microbe correlation network (Fig. 7B) further revealed extensive associations between dominant bacterial genera and multiple metabolite classes. Klebsiella showed positive correlations with several flavonoids and flavonoid glycosides, including quercetin derivatives, astragalin, diosmetin, and apigenin-related compounds, whereas Vibrio exhibited largely opposite correlation patterns with many of these metabolites. In addition, bacterial genera such as Vibrio, Pseudomonas, Secundilactobacillus, and Pediococcus were associated with various fatty acids, amino acid derivatives, and lipid-related metabolites. Studies of fermented De'ang pickled tea and green tea have reported changes in flavonoid glycoside and aglycone profiles during fermentation (Mao et al., 2024; Wang, Sun, et al., 2022). Microbial glycosidases may hydrolyze flavonoid glycosides, while the resulting aglycones may undergo further degradation, providing a plausible mechanism for changes in flavonoid composition. Nevertheless, the correlations observed here do not establish a direct role for *Klebsiella* in the synthesis or transformation of these compounds.

Overall, the multi-omics correlation analysis revealed close associations between bacterial community composition and metabolite profiles among De'ang pickled tea samples from different production years. These associations may reflect microbial contributions, shared environmental influences, or both, as differences in storage and fermentation conditions may affect microbial communities and metabolite profiles simultaneously. Controlled fermentation experiments and strain-level functional analyses are needed to establish whether the identified microorganisms directly contribute to the formation or transformation of specific metabolites.

Differences among De'ang pickled tea samples from different production years may reflect the combined effects of raw material characteristics, production conditions, and post-production storage. Variations in climatic conditions, harvesting seasons, and processing conditions may influence the initial composition of tea leaves and the subsequent development of chemical constituents, flavor profiles, and bacterial communities. Although all samples were obtained from the same manufacturer and produced according to the same enterprise protocol, year-to-year differences in raw materials and production conditions could not be completely excluded. Moreover, fresh tea leaves were not analyzed, limiting our ability to assess differences in their initial chemical and microbial characteristics. Therefore, the observed differences should be interpreted as variations among samples from different production years rather than changes attributable solely to storage duration. Future studies should include fresh tea leaves as a baseline and longitudinally monitor the same batch of tea produced under standardized conditions at multiple storage time points to distinguish production-year effects from storage-related changes and better understand their respective contributions to tea quality evolution.

## 4 Conclusion

This study integrated sensory evaluation, electronic nose analysis, chemical composition profiling, volatile compound analysis, metabolomics, bacterial community analysis, and multi-omics correlation analysis to characterize De'ang pickled tea from 12 production years (2013–2024). The results showed differences among production years in sensory characteristics, liquor color, chemical composition, metabolite profiles, volatile compounds, and bacterial communities. Eighteen major chemical constituents differed to varying degrees among production years, with more pronounced variation observed for some catechins and flavonols. Untargeted metabolomics identified 2945 metabolites and revealed distinct patterns involving flavonoid, lipid, and nitrogen-containing compound metabolism. GC–MS identified 1351 volatile compounds, including 23 key aroma compounds (rOAV ≥1), whose abundances and odor contributions varied among production years. Together, these findings provide a detailed characterization of the compositional and quality variation of De'ang pickled tea across production years.

The FRAP, DPPH, and ABTS assays demonstrated in vitro antioxidant potential, with antioxidant capacity varying among production years. Considering the widely reported antioxidant and biological activities of tea catechins and other phenolic compounds, these findings further highlight the potential health benefits and functional value of De'ang pickled tea. Microbial analysis revealed differences in bacterial diversity and community composition among production years, with Lactiplantibacillus being one of the dominant genera. Multi-omics correlation analysis further identified associations between bacterial genera and volatile and non-volatile metabolites, providing insights into the relationship between microbial communities and chemical characteristics.

Overall, De'ang pickled tea from different production years exhibited distinct patterns of quality characteristics, chemical composition, volatile profiles, antioxidant potential, and bacterial communities. These findings provide a basis for understanding production-year-related variation in De'ang pickled tea and support its quality evaluation and product characterization, while highlighting its potential health-related and functional significance.

## CRediT authorship contribution statement

**Lihong Chen:** Writing – original draft, Software, Project administration, Methodology, Investigation, Data curation. **Yunfang Li:** Visualization, Software, Formal analysis. **Xueqing Dang:** Investigation. **Fengmei Lu:** Resources. **Weixing Yang:** Investigation. **Zixian Lv:** Validation. **Yumeng Qin:** Investigation. **Yi Zhang:** Supervision, Resources. **Yan Hou:** Writing – review & editing, Project administration, Methodology, Funding acquisition, Conceptualization.

## **Funding**

This work was supported by the Major Science and Technology Special Project of Yunnan Province, China (“Key Technologies Research and Application for the Industrial Development of De'ang Pickled Tea”, 202502AE090055); Yunnan Science and Technology Talent and Platform Program (Grant No. 202405AI350130); the Key Laboratory of Agricultural Microbiome of Yunnan Province (2025RD4916CE340051).

## Declaration of competing interest

The authors declare that they have no known competing financial interests or personal relationships that could have appeared to influence the work reported in this paper.

## Data Availability

Data will be made available on request.

## Appendix A Supplementary data

Supplementary material 1: Table S1. Sensory characteristics of De’ang pickled tea from 2013 to 2024.

Supplementary material 2: Table S2. Contents of major chemical components in De’ang pickled tea from different production years.

Supplementary material 3: Table S3. Identified metabolites in De’ang pickled tea from different production years (2013–2024) based on LC–MS/MS analysis.

Supplementary material 4: Table S4. Volatile compounds identified in De’ang pickled tea samples from different production years.
